# Purification and characterization of asparaginase II from *Saccharomyces cerevisiae *cloned in *Pichia pastoris*: a study on a possible antileukemic drug

**DOI:** 10.1186/1753-6561-8-S4-P78

**Published:** 2014-10-01

**Authors:** Luciana Facchinetti de Castro Girão, Surza Lucia Gonçalves da Rocha, Ricardo Sobral Teixeira, Maria Antonieta Ferrara, Jonas Perales, Elba Pinto da Silva Bon

**Affiliations:** 1 Instituto de Química, Universidade Federal do Rio de Janeiro, 21941-909 Rio de Janeiro, Brazil; 2 Laboratório de Toxinologia, Instituto Oswaldo Cruz, Fiocruz, 21040-900 Rio de Janeiro, Brazil; 3 Instituto de Tecnologia em Fármacos, Fiocruz, 21041-250 Rio de Janeiro, Brazil

## Introduction

Bacterial asparaginase obtained from *Escherichia coli *and *Erwinia chrysanthemi *are used as medicine to treat acute lymphocytic leukemia and non-Hodgkin lymphoma. Despite the therapeutic properties of such enzymes there have been reports on adverse reactions, eventually so severe as to impede some patients of using the medicine. Besides, the only drug Brazil used to import is no longer produced. Considering these two factors our proposition is to produce non-bacterial asparaginase.

## Objective

Purify and characterize asparaginase II from *Saccharomyces cerevisiae *cloned and expressed in *Pichia pastoris *aiming to produce an antileukemic drug.

## Methodology

The established methodology to purify the recombinant asparaginase involved three steps: ultrafiltration using a 50 kDa Amicon membrane, molecular exclusion chromatography in Superdex200 and anion exchange chromatography in Mono-Q column. Homogeneity was confirmed by SDS-PAGE in reducing conditions and by MALDI-TOF/TOF mass spectrometry; enzyme activity was determined by the hydroxylaminolysis reaction. Isoelectric point was estimated by two-dimensional electrophoresis using the image analysis software ImageMaster (GE Healthcare); molecular mass was determined using 12% SDS-PAGE and molecular exclusion chromatography. The recombinant asparaginase glycidic portion in its homogeneous form was analyzed by SDS-PAGE in reducing conditions followed by periodic acid/Schiff stain. An enzymatic cleavage of N-linked oligosaccharides was performed with PNGase F at 37ºC for 3 hours and subsequent analysis by SDS-PAGE. Enzyme optimum temperature and pH were also characterized.

## Results

The homogeneous fraction obtained from the anionic exchange chromatography showed a specific activity of 204 IU mg^-1 ^which represents a purification level of 10.9 and an enzyme activity recovery of 51.3% as compared to the crude extract. After electrophoresis in reducing and denaturing conditions two bands were observed with a estimated molecular mass of 48 and 46 kDa. It was verified that these two bands correspond to asparaginase II from *S. cerevisae *and that both are glycosylated. The native enzyme molecular mass was estimated to be 136 kDa suggesting that the enzyme is an oligomer. Two optimum pH values (7.2 and 9.0) were observed. Optimum temperature at both pH, 7.2 and 9.0, was 46ºC nevertheless the activity at 37ºC was 92% of that at 46°C. After enzyme deglycosilation both bands migrated as a single one in SDS-PAGE, indicating that the main difference between them is the glycosylation level. Isoelectric point was estimated at approximately 4.55.

## Conclusion

These results support further research aiming at the use of the recombinant yeast asparaginase as an antileukemic medicine. The pre-clinic *in vitro *studies have already been initiated.

